# Penicillin G-Induced Chlamydial Stress Response in a Porcine Strain of* Chlamydia pecorum*

**DOI:** 10.1155/2016/3832917

**Published:** 2016-02-21

**Authors:** Cory Ann Leonard, Frederic Dewez, Nicole Borel

**Affiliations:** ^1^Department of Pathobiology, Institute of Veterinary Pathology, University of Zurich, Winterthurerstrasse 268, 8057 Zurich, Switzerland; ^2^University of Lille-Sciences and Technologies, Cite Scientifique, Villeneuve d'Ascq Cedex, 59655 Lille, France

## Abstract

*Chlamydia pecorum* causes asymptomatic infection and pathology in ruminants, pigs, and koalas. We characterized the antichlamydial effect of the beta lactam penicillin G on* Chlamydia pecorum* strain 1710S (porcine abortion isolate). Penicillin-exposed and mock-exposed infected host cells showed equivalent inclusions numbers. Penicillin-exposed inclusions contained aberrant bacterial forms and exhibited reduced infectivity, while mock-exposed inclusions contained normal bacterial forms and exhibited robust infectivity. Infectious bacteria production increased upon discontinuation of penicillin exposure, compared to continued exposure.* Chlamydia*-induced cell death occurred in mock-exposed controls; cell survival was improved in penicillin-exposed infected groups. Similar results were obtained both in the presence and in the absence of the eukaryotic protein translation inhibitor cycloheximide and at different times of initiation of penicillin exposure. These data demonstrate that penicillin G induces the chlamydial stress response (persistence) and is not bactericidal, for this chlamydial species/strain* in vitro*, regardless of host cell* de novo* protein synthesis.

## 1. Introduction

The family* Chlamydiaceae* comprises Gram negative, obligate intracellular bacterial pathogens of humans and wild and domestic animals. In humans,* C. trachomatis* causes trachoma and blindness as well as genital tract infection and infertility, while* C. pneumoniae* seropositivity is highly prevalent and infection can be associated with respiratory disease and atherosclerosis.* C. psittaci* and* C. abortus* are important pathogens of birds and farm animals and are capable of zoonotic transmission to humans.* C. pecorum* is generally accepted as an important pathogen of cattle, sheep, and especially koalas [1]. Symptoms associated with* C. pecorum* infection include polyarthritis, pneumonia, encephalomyelitis, and abortion in livestock and conjunctivitis, urinary/reproductive disease, and infertility in koalas [1]. In pigs,* C. pecorum* has been associated with pneumonia, enteritis, polyarthritis, pericarditis/pleuritis, and urogenital infections, as well as asymptomatic infections [2].

The* C. pecorum* strain 1710S was originally isolated in Austria from a swine abortion, in association with a large outbreak of chlamydiosis of varying clinical presentation [3, 4].* C. pecorum* 1710S experimentally infected gnotobiotic piglets demonstrated mild to moderate diarrhea and a transient stop of weight gain; these symptoms were associated with mild to moderate villus atrophy and mixed inflammatory cell infiltrates in the small intestinal mucosa [5].* C. pecorum* 1710S was also implicated, by sequence homology of cloned* Chlamydia omp1* genus-specific PCR products, in 8 of 9* Chlamydia*-positive aborted fetuses of Swiss swine [6, 7], emphasizing the pathogenic potential of this isolate. More recently, 51 female calves in the US, evaluated from birth to 6 months, all became* C. pecorum*-positive by 23S rRNA PCR and* ompA* genotyping of conjunctival and vaginal swabs;* C. pecorum* 1710S was 1 of the 3* C. pecorum* strains identified by PCR product sequencing [8]. Interestingly, in this study,* C. pecorum* infections were associated with failure to gain weight and increased conjunctival redness but not intestinal symptoms or other diseases [8]. Taken together, these data indicate that* C. pecorum* 1710S has pathogenic potential and/or can lead to decrease of animal weight gain in both swine and cattle.

In livestock, infections are routinely treated with antibiotics, especially tetracyclines [9]. However, the use of tetracyclines has been shown to be both ineffective in eradicating chlamydial infections in swine [10] and associated with the presence of tetracycline-resistant* C. suis* [11]. Beta lactam antibiotics, such as penicillin and amoxicillin, are widely used for various infections, and their persistence in animal tissue is a particular concern in meat and dairy animals, largely due to possible dangerous effects in penicillin-allergic individuals [12, 13]. In swine specifically, penicillin G has been detected in various tissues/organs, especially the kidney, for up to 39 days after injection and is expected to persist in detectable amounts for up to 47 days [14, 15].* C. pecorum* infections in pigs are frequently asymptomatic and/or are not diagnosed because they are considered unimportant pathogens in swine [2]. At the same time, the use of both veterinary-approved and off-label antibiotics is widespread in pig production. Therefore, it is likely that pigs asymptomatically infected with* C. pecorum* are treated with antibiotics administered for other purposes.

In the more well-studied Chlamydiae, such as* C. trachomatis*,* C. muridarum*,* C. pneumoniae* and* C. psittaci*, beta lactam antibiotics, such as penicillin and ampicillin, have historically and consistently been demonstrated to be ineffective against eradicating chlamydial infection, both* in vitro* and in animal models [16–22]. Instead of killing the Chlamydiae, beta lactams induce persistence or the chlamydial stress response, defined as a viable but noninfectious divergence from the normal chlamydial biphasic developmental cycle [23]. Persistent/stressed Chlamydiae are less susceptible to killing by antibiotics such as azithromycin [21, 24], undetectable by culture, and capable of resuming infectious bacteria production upon removal of the stressor/antibiotic [25].

Information about the efficacy of antibiotics against* C. pecorum* specifically, however, is very limited. A single* in vitro* study of 3 calf and lamb* C. pecorum* isolates indicated that macrolides, tetracyclines, and quinolones, but not the beta lactam ampicillin, prevented inclusion formation; however, AB induction, infectious bacterial production and potential recovery upon removal of ampicillin were not evaluated [26]. More recently, 10 koala* C. pecorum* isolates were evaluated for susceptibility to enrofloxacin, chloramphenicol, and florfenicol, but beta lactam antibiotics were not considered [27]. Thus, the ability of beta lactam antibiotics to* reversibly* abrogate* C. pecorum* infectivity, by definition inducing chlamydial stress/persistence, has not been demonstrated. The aim of our study is characterization of the* in vitro* antichlamydial effect of the beta lactam penicillin G on the* C. pecorum* porcine abortion strain 1710S, a potentially significant pathogen of swine and other economically important animals.

Our data indicate that the effect of penicillin G on* C. pecorum* 1710S is consistent with the definition of chlamydial stress/persistence. Specifically, persistence/chlamydial stress is characterized by reversible reduced or abolished infectious bacteria production and aberrant, enlarged bacterial forms called aberrant bodies (AB) detectable by immunofluorescence and transmission electron microscopy [25, 28–30]. We further determine that, under the experimental conditions used herein, penicillin G induces chlamydial stress in* C. pecorum* 1710S regardless of inclusion/exclusion of cycloheximide (which limits host* de novo* protein synthesis), though recovery is more robust in the presence of cycloheximide. Finally, initiation of penicillin exposure at 0 or 14 hours postinfection (hpi) yields equivalent reductions of infectious EB production and subsequent recovery of infectious EB production.

## 2. Materials and Methods

### 2.1. Host Cells

HeLa cells (human cervical adenocarcinoma epithelial cells, CCL-2, American Type Culture Collection, Manassas, VA, USA) were cultured for cell propagation and maintenance at 37°C and 5% CO_2_ in growth medium. Growth medium was comprised of Minimal Essential Medium (MEM) with Earle's salts, 25 mM HEPES, without L-Glutamine (GIBCO, Invitrogen, Carlsbad, CA, USA) supplemented with 10% fetal calf serum (FCS, BioConcept, Allschwil, Switzerland), 4 mM GlutaMAX-I (200 mM, GIBCO), 1% MEM Nonessential Amino Acids (100x, GIBCO), and 0.2 mg/mL gentamycin (50 mg/mL, GIBCO). For experiments, cells were seeded in 24-well plates (Techno Plastic Products AG (TPP), Trasadingen, Switzerland) at 3 × 10^5^ cells per well in 1 mL growth medium without gentamycin. Cells were seeded on 13 mm diameter glass coverslips (Sterilin Limited, Thermo Fisher Scientific, Cambridge, UK) for immunofluorescence (IF) microscopy or transmission electron microscopy (TEM) or directly in the 24-well plates, without coverslips, for titration by subpassage. Infection medium, used for inoculating cells with* Chlamydia*, consisted of all growth medium components except FCS and gentamycin. Incubation medium, used to replace infection medium after inoculation with* Chlamydia*, consisted of all growth medium components except gentamycin and was further supplemented with 1 *μ*g/mL cycloheximide (Sigma-Aldrich, St. Louis, MO, USA) immediately before use. In some experiments, growth medium without cycloheximide supplementation was used for incubation.

### 2.2. Chlamydial Strain

*C. pecorum* 1710S (isolate from a swine abortion) was kindly provided by Professor Storz, Baton Rouge, LA, USA [3] and propagated in HeLa cells. Crude* C. pecorum* stock was generated by mechanical disruption (scraping) of infected HeLa cells into infection medium, sonication (Branson Sonifier 250; Branson Ultrasonics, Danbury, CT, USA) on ice, centrifugation of infectious particles from the medium at 10,000 g at 4°C for 45 minutes, and suspension in SPG medium. Stock was stored at −80°C and frozen stock aliquots were thawed immediately before infections were carried out. SPG medium consisted of 218 mM sucrose (Sigma-Aldrich), 3.76 mM KH_2_PO_4_ (Sigma-Aldrich), 7.1 mM K_2_HPO_4_ (Merck Eurolab AG, Dietlikon, Switzerland), and 5 mM GlutaMAX-100 (GIBCO).

### 2.3. Penicillin Reagent

Penicillin G sodium salt (Sigma-Aldrich) was dissolved in sterile deionized water to a stock concentration of 20,000 units (U)/mL, filter-sterilized, and stored at −20°C. Aliquots of this penicillin G stock were thawed and further diluted in sterile water to a working concentration of 100 U/mL immediately before use. Working solutions were stored for less than one week at 4°C, as per manufacturer's recommendation. The final penicillin G concentration used in experiments, 1 U/mL, was achieved by 10 *μ*L/mL dilution of working solution in the incubation medium of penicillin-exposed samples. Sterile water (diluent) of an equivalent volume was similarly used to generate control (mock-exposed) samples.

### 2.4. Study Design, Infection, and Exposure of Infected Host Cells to Penicillin

**Figure 1:**
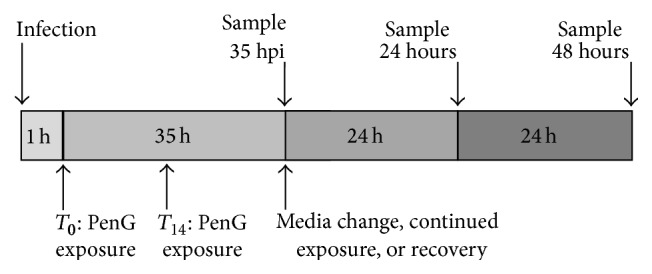
*Study Design.* The diagram illustrates infection and exposure beginning at *T*_0_, immediately postinfection, or at *T*_14_, 14 hours postinfection (hpi). HeLa cells were infected with* Chlamydia pecorum* 1710S, exposed to penicillin G (PenG) in incubation medium, and incubated until 35 hpi, at which time samples were collected for analysis. For recovery experiments, incubation medium was changed for continued PenG exposure or discontinued exposure (recovery), and samples were subjected to 24 or 48 hours (h) of additional incubation before sample collection.

HeLa cells were cultivated overnight in 24-well plates and were subsequently infected with* C. pecorum* 1710S at 1 multiplicity of infection (MOI) in 1 mL infection medium per well and centrifuged for 1 hour (h) at 1000 g and 25°C. After centrifugation, infection medium was immediately replaced with incubation medium and cultures were incubated at 37°C and 5% CO_2_ as previously described [31]. The crude stocks used resulted in approximate control infection rates of 50%; this corresponded to approximately 10^7^ recoverable inclusion-forming units (IFU) per well for mock-exposed infected cells after 35 hours of incubation. The effect of penicillin G on* C. pecorum* inclusions was determined by adding penicillin G to a final concentration of 1 U/mL, to the incubation medium of infected cells at *T*_0_, immediately after infection, or at *T*_14_, 14 hours after infection (Figure 1). In experiments evaluating recovery from penicillin G, incubation medium was changed at 35 hours postinfection (hpi) to continue exposure or discontinue exposure (recovery) to penicillin. This time, 35 hpi, corresponds to the presence of mature* C. pecorum* 1710S inclusions in HeLa culture, but no significant host cell lysis, as previously reported [32].

At the indicated times, samples were collected and processed for further analysis as previously described [31]. For immunofluorescence (IF) microscopy, cells were fixed with absolute methanol (−20°C) for 10 minutes. For transmission electron microscopy (TEM), cells were fixed with 2.5% glutaraldehyde (Electron Microscopy Sciences, Fort Washington, USA) for 1 h and embedded in epoxy resin (Fluka; Sigma-Aldrich) by routine methods. For titration by subpassage infected monolayers collected at 35 hpi were scraped into 1 mL of fresh infection medium and stored at −80°C. Because* C. pecorum* 1710S-infected HeLa cells undergo significant lysis and EB release soon after 35 hpi, as previously reported [32], for continued exposure or recovery sample collection for titration by subpassage, infected monolayers were scraped into the* existing* incubation medium and stored at −80°C. In all experiments, biological duplicates were generated for each experimental condition.

### 2.5. IF Microscopy

*C. pecorum* inclusions were visualized using 1 : 200 diluted* Chlamydiaceae *family-specific mouse monoclonal antibody directed against the chlamydial lipopolysaccharide (LPS, Clone ACI-P; Progen, Heidelberg, Germany) and 1 : 500 diluted Alexa Fluor 488-conjugated secondary goat anti-mouse antibody (Molecular Probes, Eugene, OR, USA). Host and chlamydial DNA were labeled with 1 *μ*g/mL 4′,6-diamidino-2′-phenylindole dihydrochloride (DAPI, Molecular Probes). Coverslips were mounted with FluoreGuard mounting medium (Hard Set; ScyTek Laboratories Inc., Logan, UT, USA) on glass slides. Slides were evaluated using a Leica DMLB fluorescence microscope (Leica Microsystems, Wetzlar, Germany) under oil immersion at 1000x magnification with a 1006 objective (PL FLUOTAR 100x/1.30, OIL, ∞/0.17/D, Leica Microsystems) and a 106 ocular objective (Leica L-Plan 10x/25 M, Leica Microsystems). To determine percent of cells infected (inclusions per nucleus) and mean number of nuclei per field (to evaluate possible cell loss from the monolayer), HeLa nuclei and corresponding chlamydial inclusions in each of 10 randomly selected microscopic fields were counted per duplicate coverslip (at least 200 HeLa nuclei per coverslip), and the mean of coverslips values was generated for each experimental condition. Representative microscopic images were captured using BonTec software (BonTec) and a UI-2250SE-C-HQ camera (uEye, IDS Imaging Development Systems GmbH, Obersulm, Germany).

### 2.6. Chlamydial Titration by Subpassage

HeLa cells were grown on glass coverslips as described above. Immediately prior to use, the previously prepared and frozen samples were thawed and sonicated on ice for 5 minutes. Sonicated samples were serially diluted in infection medium on the prepared HeLa cells. Centrifugation, infection medium replacement with incubation medium (containing 1 *μ*g/mL cycloheximide for all experiments), and incubation were carried out as described for infection of host cells. Fixation and immunostaining were performed exactly as described for IF microscopy. The number of inclusions in 30 random microscopic fields per duplicate coverslip was counted using a Leica fluorescence microscope at 200x magnification with a 206 objective (PL FLUOTAR 20x/0.50 PH 2, ∞/0.17/B) and a 106 ocular objective (Leica L-Plan 10x/25 M, Leica Microsystems), and the mean of coverslips values was generated for each experimental condition. IFU/mL of undiluted inoculum (representing IFU per total collected material from a single well of a 24-well plate) was calculated according to previously published methods [33].

### 2.7. TEM

Ultrathin (80 nm) sections were mounted on gold grids (Merck) and contrasted with uranyl acetate dehydrate (Fluka; Sigma-Aldrich) and lead citrate (Merck). Sections were subsequently evaluated using a Philips CM10 electron microscope (Software release version 5.1; FEI Company, Hillsboro, OR, USA) and imaged using a Gatan Orius SC 1000 CCD Camera with software version Digital Micrograph 2.30 (Gatan Inc., Warrendale, PA, USA). All images were analyzed using Photoshop CS6 software (Adobe Systems Incorporated, San Jose, CA, USA). To determine the presence of AB, bacterial morphology analysis was carried out, as previously described [34]: EB (dark, 0.25–0.5 *μ*m), IB (intermediate bodies; dark center and pale periphery, equivalent in size to EB or RB), RB (pale, 0.5–1 *μ*m), and AB (pale, ≥2 *μ*m).

### 2.8. Statistical Analysis

In all experiments, biological duplicates were averaged to generate results. Statistical analyses were performed using Microsoft Excel. Significance of the difference of means was determined by unpaired *t*-test and *p* values of <0.05 were considered significant. *p* values were confirmed using the GraphPad QuickCalcs Web site: http://www.graphpad.com/quickcalcs/ttest1/ (accessed June 2014). Unless stated otherwise, results are displayed as means ± standard deviation, of the results from 2 or 3 independent experiments. In cases where a single confirmatory experiment is noted, results are displayed as means ± standard deviation, of the two biological duplicates.

## 3. Results and Discussion

In this study we evaluated the effect of the beta lactam antibiotic penicillin G on a porcine abortion strain of* Chlamydia pecorum* (1710S). Based on previous reports that various chlamydial species enter chlamydial stress/persistence upon beta lactam exposure [16–22], we hypothesized that penicillin G would induce chlamydial stress in* C. pecorum* 1710S. Because inclusion/exclusion of the eukaryotic protein synthesis inhibitor cycloheximide has the potential to influence the effect of beta lactams on Chlamydiae [35], we evaluated the effect of penicillin G on* C. pecorum* in the presence of cycloheximide and performed a single confirmatory experiment in the absence of cycloheximide.

**Figure 2:**
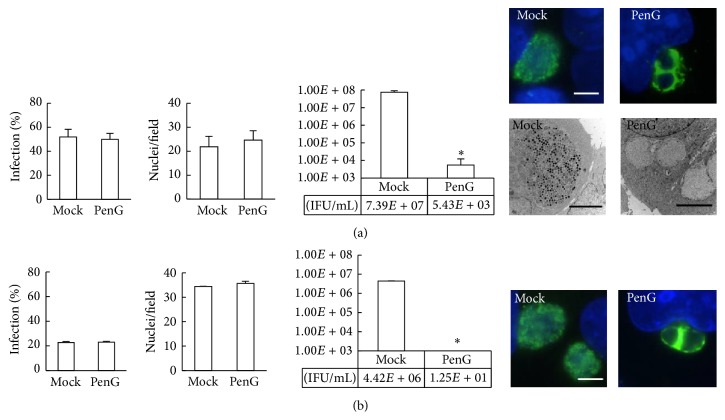
Penicillin G exposure induces aberrant body formation and reduces infectious elementary body production in* Chlamydia pecorum*.* C. pecorum*-infected HeLa cells were exposed to penicillin G (PenG), at 0 hours postinfection (hpi) until 35 hpi. Control infected cells were exposed to diluent only (mock). Cycloheximide was included (a) or not included (b) in the incubation medium. Inclusions were visualized using an antibody directed against chlamydial lipopolysaccharide (green), and DNA was labeled with 4′,6-diamidino-2′-phenylindole dihydrochloride (blue). Percent of host cells infected and mean nuclei per field was determined by evaluation of 10 fields (≥200 cells), per coverslip, per sample. Representative immunofluorescence and transmission electron microscopic images illustrate normal inclusions in mock-exposed controls and inclusions containing aberrant bodies upon penicillin exposure. Infectious elementary body production was determined by titration by subpassage and expressed as inclusion-forming units (IFU)/mL. Results are means ± standard deviation. The two-tailed* t*-test was used to compare means;* p* ≤ 0.05 = significant (^*∗*^); *n* = 3 (a) or *n* = 2 (b). Scale bars = 5 *μ*m.

HeLa cells were infected with* C. pecorum*, exposed to penicillin G or sterile water (mock-exposed control) in the cycloheximide-containing or cycloheximide-free incubation medium at 0 hours postinfection (hpi), and incubated until 35 hpi (Figure 1). For cycloheximide-exposed HeLa/*C. pecorum* (Figure 2(a)), infection rates of 50–52% of diluent- or 0 hpi penicillin-exposed cells were observed. Mean nuclei per field values were 22 and 25 for diluent- and 0 hpi penicillin-exposed infected cells, respectively. When cycloheximide was omitted (Figure 2(b)), an infection rate of 23% was observed for mock- and 0 hpi penicillin-exposed cells. In the absence of cycloheximide, mean nuclei per field values were 35 for mock-exposed cells and 36 for 0 hpi penicillin-exposed cells. Thus, penicillin G had no effect on host cell infection rate or host cell loss from the monolayer at 35 hpi, regardless of time of initiation of penicillin exposure, whether or not cycloheximide was included. However, as expected, the omission of cycloheximide allowed host cell division to continue and resulted in increased nuclei per field compared to cycloheximide-containing cultures. Additionally, infection rates in the presence of cycloheximide were approximately twice those observed in the absence of cycloheximide.

By IF microscopy, AB were easily observed in all inclusions in 0 hpi penicillin-exposed infected cells, whether cycloheximide was included (Figure 2(a)) or excluded (Figure 2(b)). Corresponding mock-exposed infected cells contained no AB, regardless of the inclusion/exclusion of cycloheximide. Confirmatory TEM analysis (Figure 2(a)) demonstrated that the AB observed by IF microscopy were at least 2 *μ*m in diameter, as is typical for AB, and that mock-exposed controls included EB and RB of normal size and morphology [34]. Titration by subpassage was used to evaluate infectious EB production, measured as inclusion-forming units (IFU) per mL. Mock-exposed inclusions cultured in the presence of cycloheximide yielded 7.39 × 10^7^ IFU/mL (per well of cells), while 0 hpi penicillin-exposed inclusions yielded 5.43 × 10^3^ IFU/mL (Figure 2(a)). In the absence of cycloheximide, mock-exposed inclusions yielded 4.42 × 10^6^ IFU/mL, while 0 hpi penicillin-exposed inclusions yielded 1.25 × 10^1^ IFU/mL (Figure 2(b)). Inclusion of cycloheximide resulted in increased IFU/mL in mock-exposed controls compared to exclusion of cycloheximide, as expected given the increased infection rate of host cells also observed. However, the observed penicillin-dependent decreases in IFU/mL constituted a similar reduction to <1% of the mock-exposed control whether or not cycloheximide was included.

**Figure 3:**
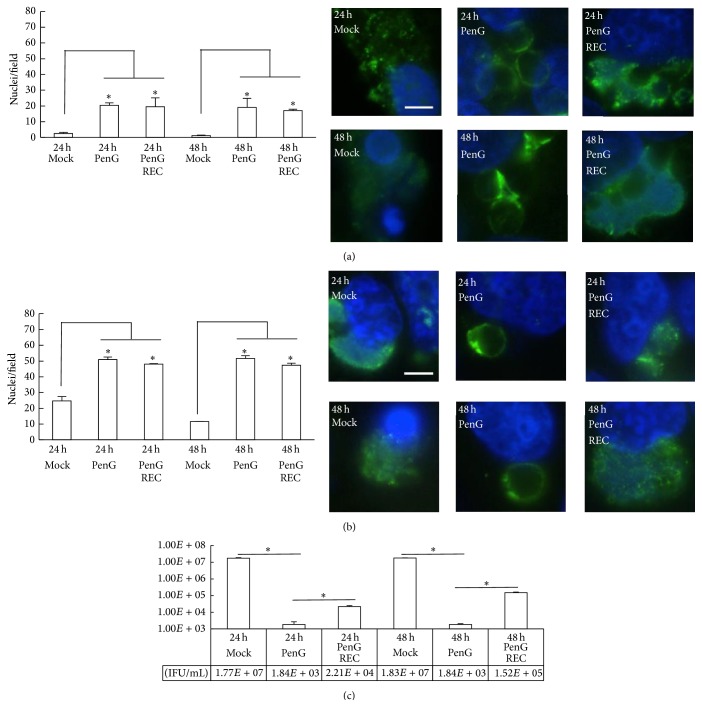
Penicillin G exposure prevents* Chlamydia pecorum*-mediated cell death and* C. pecorum* recovers infectivity upon discontinuation of penicillin exposure.* C. pecorum*-infected HeLa cells were penicillin G (PenG) exposed or diluent exposed (mock) from 0 to 35 hours postinfection (hpi). Cycloheximide was included (a) or not included (b, c) in the incubation medium. At 35 hpi, medium was changed to continue or discontinue (recovery, REC) penicillin exposure for 24 or 48 hours (h) and cycloheximide was maintained for (a). Inclusions were visualized and mean nuclei per field were determined as described for Figure 2. Representative immunofluorescence microscopic images illustrate cell death in mock-exposed controls, inclusions containing aberrant bodies upon continued penicillin exposure and normal-appearing inclusions upon recovery (REC). Production of infectious elementary bodies was determined by titration by subpassage and expressed as inclusion-forming units (IFU)/mL. Results are means ± standard deviation. The two-tailed* t*-test was used to compare means;* p* ≤ 0.05 = significant (^*∗*^); *n* = 2. Scale bars = 5 *μ*m.

To determine the ability of* C. pecorum* to recover from penicillin exposure, infected cells were exposed at 0 hpi until 35 hpi, when the medium was changed and exposure was continued or discontinued (recovery) for 24 h or 48 h (Figure 1). Cycloheximide exposure was concomitantly continued (Figure 3(a)) or excluded (Figures 3(b) and 3(c)) for the duration of incubation. Upon 24 h or 48 h of continued culture, significant* Chlamydia*-induced cell death occurred in mock-exposed controls, while cell survival was comparatively improved in 0 hpi penicillin-exposed groups, regardless of inclusion/exclusion of cycloheximide. In the presence of cycloheximide, mean nuclei per field values ranged from 17 to 20 for 0 hpi penicillin-exposed cells, upon both 24 h and 48 h of continued culture, while mock-exposed controls retained only 1–3 nuclei per field (Figure 3(a)). In the absence of cycloheximide, mean nuclei per field values were larger and ranged from 47 to 52 for 0 hpi penicillin-exposed cells at 24 and 48 h of further culture, while mock-exposed cells retained 12–25 nuclei per field (Figure 3(b)). Therefore, for 24 h and 48 h continued culture, penicillin G exposure significantly protected HeLa cells from* C. pecorum* 1710S-induced cell death independent of cycloheximide inclusion/exclusion. Thus, cells remained present in both cycloheximide-exposed and unexposed HeLa monolayers as a source of constituents/metabolites for potentially recovering Chlamydiae.

Infectious EB production (IFU/mL) increased upon discontinuation of 0 hpi-initiated penicillin exposure, compared to continued penicillin exposure, for both cycloheximide-exposed infected cells (Table 1) and unexposed HeLa/*C. pecorum* (Figure 3(c)). Although the statistical significance of these changes was inconsistent due to high variability, the observed changes were generally substantial. Approximately 600- to 5000-fold increases for 0 hpi penicillin exposure were observed when comparing discontinued exposure (recovery) compared to continued penicillin exposure, in the presence of cycloheximide, at 24 h of continued culture (Table 1). Similarly, but to a greater extent, approximately 30,000- to 340,000-fold increases in IFU/mL of 0 hpi penicillin-exposed groups were observed in cycloheximide-including recovery conditions, compared to continued exposure upon 48 h of continued culture (Table 1). In the absence of cycloheximide, results were similar but more moderate in nature. At 24 h of continued culture, discontinuation of penicillin exposure resulted in a 12-fold increase in IFU/mL compared to continued exposure, while, at 48 h of continued culture, an 82-fold increase in IFU/mL was observed in discontinued penicillin exposure versus continued exposure (Figure 3(c)). Thus, the inclusion of cycloheximide potentiated a more robust recovery from penicillin G exposure. However, either inclusion or exclusion of cycloheximide allowed increased infectious* C. pecorum* EB production upon discontinuation of penicillin exposure. Furthermore, regardless of the inclusion/exclusion of cycloheximide, increased recovery time from 24 h to 48 h of continued culture was associated with increased infectious EB production.

AB were consistently observed by IF microscopy upon continued penicillin exposure, regardless of the duration of exposure or the presence (Figure 3(a)) or absence (Figure 3(b)) of cycloheximide in the assay. Some, but not all, recovery group inclusions showed areas of denser, more granular LPS-positive staining, consistent with the appearance of mock-exposed inclusions at 35 hpi (Figures 3(a) and 3(b) right panels, compared to Figures 2(a) and 2(b) left panels). These inclusions were frequently associated with shrunken, brightly staining host cell nuclei, indicating that as inclusions became more normal in appearance,* C. pecorum* 1710S was still capable of exerting its cytotoxic effect on HeLa cells. Mock-exposed cells showed no AB, at 24 h or 48 h of continued culture, regardless of inclusion/exclusion of cycloheximide or duration of incubation (Figures 3(a) and 3(b)).

Initiation of penicillin G exposure at 14 hpi (see Figure 1), in the presence of cycloheximide, was carried out to determine if Chlamydiae being at the stage of early RB development upon exposure might modulate the described effects of penicillin G exposure or subsequent continuation/discontinuation of this exposure on Chlamydiae. During 0 hpi penicillin G exposure, EB are exposed to penicillin as they enter the host cells and initiate inclusion formation. However, 14 hpi addition of penicillin postpones the exposure of the Chlamydiae until EB have differentiated into RB, and early RB replication/development is starting or will soon start, but redifferentiation into EB has not begun [36]. Thus, upon *T*_14_ exposure, intracellular RB are the bacterial forms initially exposed to penicillin.

We found, by IF microscopy and TEM (not shown), that 14 hpi penicillin G-exposed HeLa/*C. pecorum* had normal inclusions in mock-exposed groups and AB-containing inclusions in 14 hpi penicillin-exposed groups at 35 hpi and at 24 h or 48 h of continued exposure, similar to the 0 hpi penicillin exposure results. Later initiation of exposure was associated with somewhat larger numbers (usually 6 to 8 AB, versus 1-2 AB per inclusion) of slightly smaller AB (though still at least 2 *μ*m in diameter). Upon 14 hpi penicillin exposure, the mean nuclei per field value was 24 and the host cell infection rate was 51% at 35 hpi. For 14 hpi initiation of penicillin exposure and subsequent continuation or discontinuation of penicillin exposure, mean nuclei per field values at 24 h and 48 h of continued culture ranged from 16 to 22 and 15 to 16, respectively. These 35 hpi and 24/48 h data are statistically equivalent to those observed for 0 hpi penicillin exposure. Additionally, upon 14 hpi initiation of penicillin exposure, the IFU/mL value at 35 hpi (2.08 × 10^4^ IFU/mL) was less than 1% of the mock-exposed control value, similar to the 0 hpi results. Furthermore, IFU/mL values of discontinued penicillin exposure versus continued penicillin exposure (see Table 1) were very similar regardless of time of initiation of penicillin exposure. Later initiation of exposure, and thus decreased total penicillin exposure time, was not correlated with increased infectious EB production (IFU/mL).

Beta lactams have long been considered nonbactericidal against the Chlamydiae, instead inducing,* in vitro*, a reversible state of bacterial stress, termed persistence or chlamydial stress. The original report of* in vitro* chlamydial persistence [16] described aberrant bacterial forms and reduced infectivity upon penicillin G exposure and demonstrated the reversibility that defines chlamydial persistence, a phenomenon now known to be inducible by various stressors including nutrient deprivation, host interferon gamma production, and coinfection [30]. The* in vitro* antichlamydial effect of beta lactams has also been reported as bactericidal under some conditions, namely, exposure to penicillin early after chlamydial infection and specifically in the absence of pharmacologically (cycloheximide) inhibited host* de novo* protein synthesis [35]. Chlamydiae were thought to lack peptidoglycan, the target of beta lactams, so the response of Chlamydiae to beta lactams has been anomalous [37]. A recent report providing evidence of chlamydial peptidoglycan [38] clarifies the antichlamydial effect of beta lactams and emphasizes the role they may play in modulation of natural infections.

In this study, experiments were primarily designed to facilitate* C. pecorum* recovery from penicillin to determine if this species can, under conditions permissive for persistence induction, exhibit the hallmarks of persistence. In a previous report, exclusion of cycloheximide was sufficient to render* C. trachomatis* incapable of recovery from 1, 10, or 100 U/mL penicillin G exposure, while recovery from 100 U/mL penicillin G was possible in the presence of cycloheximide [35]. Thus, we included cycloheximide in our experiments but also compared the effects to those elicited in the absence of cycloheximide. We found that although cycloheximide potentiated the recovery of* C. pecorum* 1710S infectious EB production upon the removal of penicillin G, it was not required for such recovery.* In vivo*, local modulation of host protein synthesis may occur via mechanisms in response to chlamydial infection and/or infection with other bacteria or viruses. These may include immune-mediated interferon-dependent tryptophan reduction [29] or specific antiviral responses [39], for example. Therefore, though cycloheximide may be considered to limit the biological relevance of* in vitro* models of chlamydial infection, reduced host protein translation may impact chlamydial entry into and exit from the chlamydial stress response* in vivo*.

HeLa cells, being human-derived cells, do not represent a natural host species for* C. pecorum*. However, previous reports indicate that penicillin G exposure of* C. trachomatis*-infected HeLa cells, both in the presence [35] and the absence [22] of cycloheximide, induces an antichlamydial effect consistent with persistence. Additionally, recent work in our laboratory [32] showed that damage associated molecular patterns (DAMP) exposure of* C. pecorum*-infected cells also elicits an antichlamydial effect in HeLa cells that is consistent with persistence.

The concentration of penicillin G used in this study was chosen based on preliminary optimization (not shown) of the induction of AB formation in various chlamydial species and host cells lines, in which 1–100 U/mL consistently yielded AB in all inclusions. While studies evaluating penicillin-induced persistence frequently use hundreds of U/mL, a recent* in vitro* study showed penicillin G reduced* C. trachomatis* infectious EB production by >99% at 0.02 U/mL [22]. Thus, we chose a concentration not in substantial excess of that required to induce* C. pecorum* 1710S AB formation and to reduce infectious EB production. 1 U/mL penicillin G induced AB formation in all inclusions and reduced infectious EB production to <1% of control values. Earlier exposure did not result in statistically reduced infectious EB production. Furthermore, later exposure, resulting in more AB per inclusion but a similar number of inclusions compared to early exposure or mock exposure, did not increase recovery of infectious EB production upon cessation of penicillin exposure.

Chlamydial species is also likely to impact the effect of beta lactams both* in vitro* and* in vivo*. We recently demonstrated that* C. pecorum* is more capable of recovering from DAMP-induced reduced infectious EB production than* C. trachomatis* [32]; this suggests that* C. pecorum* may be more able to recover from persistence induced by various stressors than* C. trachomatis*. Human strains of* C. pneumoniae* are more able to recover from penicillin-induced persistence than animal strains [40], suggesting that species- and strain-specific sensitivity to stressors, including antibiotics, may be common within the Chlamydiae. Experimental parameters such as specific beta lactam drug used, timing of initiation exposure and duration of exposure, and timing of removal of drug for recovery and duration of recovery, as well as differences in measurement/determination of infectivity could be expected to impact degree and detection of entry into and recovery from beta lactam-induced chlamydial stress/persistence. Such factors, as well as biological variation amongst the Chlamydiae, are also likely to contribute to differing reports of bactericidal and bacteriostatic effects of beta lactam antibiotics amongst the Chlamydiae.

Questions remain concerning the biological importance of chlamydial persistence/stress* in vivo*, especially in naturally occurring infections. While aberrant chlamydial forms have been demonstrated in porcine [41] and human [42] specimens, veterinary/medical consequences are debated [23]. Results from a recent mouse model study demonstrated that beta lactam-induced persistent chlamydial genital infection is more prone to azithromycin treatment failure than productive chlamydial infection [21]. Intestinal chlamydial infection in mice has also been demonstrated to be more resistant to azithromycin than genital infection [43]. Asymptomatic gastrointestinal* C. pecorum* infections and other such chlamydial infections are common and are subject to beta lactam exposure due to widespread agricultural use of these drugs. Thus, beta lactam therapy is likely to expose undetected Chlamydiae to persistence inducers. While it is known that persistent/stressed Chlamydiae exhibit altered protein expression* in vitro* compared to nonstressed controls [44–48], the potential effect of AB/chlamydial stress on porcine abortion rates, failure to gain weight, or other clinical symptoms is entirely unknown but interesting and potentially important.

Recent clinical findings suggest that asymptomatic human gastrointestinal chlamydial infection also occurs [49–51]. These data and clinical findings suggest that difficulties in elimination of chlamydial infection may be common to both humans and other animals and may be modulated by beta lactam exposure. In a recent porcine-specific example of failure to eliminate chlamydial infection with standard antimicrobial therapy, asymptomatic chlamydial intestinal infection in multiple pig herds in Switzerland remained present independent of antibiotic treatments, including tetracycline treatment [10].

## 4. Conclusions

We report that, as hypothesized,* C. pecorum* 1710S enters a state of chlamydial/stress persistence upon beta lactam exposure. However, the extensive biological and genetic diversity of* C. pecorum*, including various clinical presentations and serological characteristics,* in vitro* growth characteristics, and genetic characteristics such as the recently demonstrated differential presence of a novel plasmid [4, 52–54], suggests that the wide range of strains comprising this species warrant continued scrutiny. Since the effect of asymptomatic chlamydial infections on long-term fitness of animals and humans and the role of chlamydial stress/persistence under such circumstances is unknown, the effects of beta lactams on the Chlamydiae are of continued veterinary and medical interest.

## Figures and Tables

**Table 1:** *Chlamydia pecorum* recovers infectious elementary body production (inclusion-forming units/mL) upon discontinuation of penicillin G exposure.

Hours	Experimental group	Experiment 1	*p*	Experiment 2	*p*
24	Mock	1.21 × 10^8^ ± 2.20 × 10^6^		5.11 × 10^7^ ± 1.21 × 10^6^	
	PenG 0 hpi	3.25 × 10^1^ ± 1.77 × 10^1^	0.0003^**#**^	1.00 × 10^1^ ± 7.07 × 10^0^	0.0002^**#**^
	PenG 0 hpi recovery	1.77 × 10^5^ ± 1.00 × 10^4^	0.0016^**##**^	6.36 × 10^3^ ± 2.75 × 10^3^	0.0825^**##**^
	PenG 14 hpi	1.75 × 10^1^ ± 3.54 × 10^0^	0.0003^**#**^	3.00 × 10^1^ ± 1.41 × 10^1^	0.0002^**#**^
	PenG 14 hpi recovery	1.63 × 10^5^ ± 1.40 × 10^4^	0.0037^**##**^	1.52 × 10^4^ ± 2.13 × 10^4^	0.4196^**##**^

48	Mock	4.89 × 10^7^ ± 2.73 × 10^7^		2.73 × 10^7^ ± 9.06 × 10^5^	
	PenG 0 hpi	5.50 × 10^1^ ± 2.83 × 10^1^	0.0026^**#**^	3.00 × 10^1^ ± 7.07 × 10^0^	0.0019^**#**^
	PenG 0 hpi recovery	1.66 × 10^6^ ± 1.18 × 10^5^	0.1345^**##**^	1.02 × 10^7^ ± 5.62 × 10^5^	0.0015^**##**^
	PenG 14 hpi	1.28 × 10^2^ ± 3.54 × 10^0^	0.1314^**#**^	6.75 × 10^1^ ± 3.54 × 10^0^	0.0249^**#**^
	PenG 14 hpi recovery	9.69 × 10^5^ ± 6.02 × 10^3^	<0.0001^**##**^	6.11 × 10^6^ ± 4.73 × 10^6^	0.2091^**##**^

*C. pecorum *1710S-infected HeLa cells were exposed to diluent only (mock) or to penicillin G at 0 hours postinfection (hpi) or 14 hpi. At 35 hpi, medium was changed and exposure was continued or discontinued (recovery) for 24 or 48 hours. Inclusion counts generated from biological duplicates were used to calculate mean inclusion-forming units/mL. Results from 2 independent experiments evaluating 24 h of recovery or 48 h of recovery are shown. *p* values for groups compared to corresponding mock (**#**) or corresponding continued exposure groups (**##**) are shown; *p* ≤ 0.05 = significant.
